# Supplementary material for: Noise-invariant representations of sound emerge along the canonical cortical hierarchy
Source: PLoS Biol. 2026 Jul 20;24(7):e3003915. doi: 10.1371/journal.pbio.3003915 (PMC13399537; doi:10.1371/journal.pbio.3003915)
Supplement: S1 Table — (PDF) [file pbio.3003915.s005.pdf]

S1 Table: Per-FOV slope and bias modulation proportions for frequency tuning curves.

Cell Type	Mouse ID / FOV		Slope			Bias	
			Proportion	# Sig.		Proportion	# Sig.
L2/3	Emx045_1	mult.	0.500	6	add.	0.625	8
		div.	0.500		sub.	0.375	
	Emx047_1	mult.	0.667	9	add.	0.333	3
		div.	0.333		sub.	0.667	
	Emx077_1	mult.	0.400	50	add.	0.476	21
		div.	0.600		sub.	0.524	
	Emx077_2	mult.	0.357	14	add.	0.353	17
		div.	0.643		sub.	0.647	
	Emx079_1	mult.	0.222	18	add.	0.600	15
		div.	0.778		sub.	0.400	
	Emx079_2	mult.	0.520	25	add.	0.526	19
		div.	0.480		sub.	0.474	
	Tlx209_1	mult.	0.484	31	add.	0.591	22
		div.	0.516		sub.	0.409	
	Tlx209_2	mult.	0.091	11	add.	0.600	5
		div.	0.909		sub.	0.400	
	WT348_1	mult.	0.082	61	add.	0.300	30
		div.	0.918		sub.	0.700	
	WT350_1	mult.	0.000	47	add.	0.143	21
		div.	1.000		sub.	0.857	
L5 IT	Tlx194_1	mult.	0.629	35	add.	0.800	15
		div.	0.371		sub.	0.200	
	Tlx194_2	mult.	0.719	32	add.	0.500	12
		div.	0.281		sub.	0.500	
	Tlx194_3	mult.	0.267	30	add.	0.688	16
		div.	0.733		sub.	0.313	
	Tlx457_1	mult.	0.903	31	add.	0.500	26
		div.	0.097		sub.	0.500	
	Tlx471_1	mult.	0.700	10	add.	0.600	10
		div.	0.300		sub.	0.400	
	Tlx477_1	mult.	0.128	39	add.	0.444	18
		div.	0.872		sub.	0.556	
	Tlx477_3	mult.	0.185	27	add.	0.438	16
		div.	0.815		sub.	0.563	
	Tlx479_1	mult.	0.071	14	add.	0.375	8
		div.	0.929		sub.	0.625	
	Tlx479_2	mult.	0.333	6	add.	0.250	4
		div.	0.667		sub.	0.750	

Continued on next page

Table S1 continued

Cell Type	Mouse ID / FOV		Slope			Bias	
			Proportion	# Sig.		Proportion	# Sig.
	Tlx495_1	mult.<br>div.	0.250<br>0.750	20	add.<br>sub.	0.000<br>1.000	3
	Tlx495_2	mult.<br>div.	0.357<br>0.643	14	add.<br>sub.	1.000<br>0.000	6
L5 ET	ET402_1	mult.<br>div.	0.591<br>0.409	22	add.<br>sub.	0.583<br>0.417	12
	ET747_1	mult.<br>div.	0.480<br>0.520	25	add.<br>sub.	0.688<br>0.313	16
	ET749_1	mult.<br>div.	0.429<br>0.571	14	add.<br>sub.	0.500<br>0.500	8
	ET767_1	mult.<br>div.	0.278<br>0.722	18	add.<br>sub.	0.909<br>0.091	11
	ET767_2	mult.<br>div.	0.222<br>0.778	9	add.<br>sub.	1.000<br>0.000	2
