# Supplementary material for: Noise-invariant representations of sound emerge along the canonical cortical hierarchy
Source: PLoS Biol. 2026 Jul 20;24(7):e3003915. doi: 10.1371/journal.pbio.3003915 (PMC13399537; doi:10.1371/journal.pbio.3003915)
Supplement: S2 Table — (PDF) [file pbio.3003915.s006.pdf]

S2 Table: Per-FOV slope and bias modulation proportions for intensity tuning curves.

Cell Type	Mouse ID / FOV		Slope			Bias	
			Proportion	# Sig.		Proportion	# Sig.
L2/3	Emx045_1	mult.	0.625	8	add.	0.500	6
		div.	0.375		sub.	0.500	
	Emx047_1	mult.	0.571	7	add.	0.333	3
		div.	0.429		sub.	0.667	
	Emx077_1	mult.	0.410	39	add.	0.588	17
		div.	0.590		sub.	0.412	
	Emx077_2	mult.	0.300	10	add.	0.625	8
		div.	0.700		sub.	0.375	
	Emx079_1	mult.	0.214	14	add.	0.222	9
		div.	0.786		sub.	0.778	
	Emx079_2	mult.	0.611	18	add.	0.667	6
		div.	0.389		sub.	0.333	
	Tlx209_1	mult.	0.467	30	add.	0.773	22
		div.	0.533		sub.	0.227	
	Tlx209_2	mult.	0.222	9	add.	0.500	2
		div.	0.778		sub.	0.500	
	WT348_1	mult.	0.140	57	add.	0.333	18
		div.	0.860		sub.	0.667	
	WT350_1	mult.	0.025	40	add.	0.125	16
		div.	0.975		sub.	0.875	
L5 IT	Tlx194_1	mult.	0.655	29	add.	0.867	15
		div.	0.345		sub.	0.133	
	Tlx194_2	mult.	0.842	19	add.	0.556	9
		div.	0.158		sub.	0.444	
	Tlx194_3	mult.	0.353	17	add.	0.600	10
		div.	0.647		sub.	0.400	
	Tlx457_1	mult.	0.880	25	add.	0.438	16
		div.	0.120		sub.	0.563	
	Tlx471_1	mult.	0.857	7	add.	0.500	4
		div.	0.143		sub.	0.500	
	Tlx477_1	mult.	0.219	32	add.	0.692	13
		div.	0.781		sub.	0.308	
	Tlx477_3	mult.	0.211	19	add.	0.333	15
		div.	0.789		sub.	0.667	
	Tlx479_1	mult.	0.901	11	add.	0.250	4
		div.	0.909		sub.	0.750	
	Tlx479_2	mult.	0.571	7	add.	1.000	2
		div.	0.429		sub.	0.000	

Continued on next page

Table S2 continued

Cell Type	Mouse ID / FOV		Slope			Bias	
			Proportion	# Sig.		Proportion	# Sig.
	Tlx495_1	mult.<br>div.	0.231<br>0.769	13	add.<br>sub.	0.333<br>0.667	3
	Tlx495_2	mult.<br>div.	0.385<br>0.615	13	add.<br>sub.	1.000<br>0.000	4
L5 ET	ET402_1	mult.<br>div.	0.667<br>0.333	21	add.<br>sub.	0.692<br>0.308	13
	ET747_1	mult.<br>div.	0.571<br>0.429	21	add.<br>sub.	0.571<br>0.429	14
	ET749_1	mult.<br>div.	0.286<br>0.714	7	add.<br>sub.	0.600<br>0.400	5
	ET767_1	mult.<br>div.	0.263<br>0.737	19	add.<br>sub.	0.400<br>0.600	10
	ET767_2	mult.<br>div.	0.286<br>0.714	7	add.<br>sub.	0.000<br>1.000	1
